# Parental history of dementia does not confound the association between depression and decline in cognitive status

**DOI:** 10.1002/alz.090356

**Published:** 2025-01-09

**Authors:** Chad Guilliams, Erin L. Abner

**Affiliations:** ^1^ University of Kentucky, Lexington, KY USA; ^2^ Department of Epidemiology, University of Kentucky, Lexington, KY USA

## Abstract

**Background:**

Parental history of dementia may lead to participation bias and has been largely replaced in analyses with genetic markers such as *APOEe4*. We studied how the inclusion of parental history of dementia affects associations between depression and cognitive status.

**Method:**

Participants without dementia were stratified by cognitive and depression status at their initial study visit (“baseline”): normal cognition without depression (*n = 7,592*), normal cognition with depression (*n = 2,346*), MCI without depression (*n = 2,280*), and MCI with depression (*n = 1,525*). Logistic regression and Fine and Gray models, with death of the participant treated as a competing event, were used to analyze whether parental history of dementia affects the association between depression and cognitive status at baseline (*n = 20,796*) and over time. Fine and Gray models were used for participants with normal cognition at baseline and then again for NACC participants with MCI at baseline to estimate associations for the conversion to MCI or dementia respectively. Models included *APOEe4* dose, participant demographics (age, sex, race, and education), in addition to self‐reported depression and depressive symptoms.

**Result:**

The adjusted odds of MCI were increased for those with depression at baseline, OR = 2.51 (95% CI: 2.36‐2.68), and inclusion of parental history of dementia in the logistic regression analysis slightly strengthened the association (OR = 2.55 [2.39‐2.73]). Selection bias was evident, as participants reporting both parents with dementia had the lowest adjusted odds of cognitive impairment (OR 0.65, 95% CI: 0.57‐0.73) compared to no reported parental history. In the competing risk analysis, among those with normal cognition at baseline, those with depression had an increased risk of converting to MCI or dementia, subdistribution hazard ratio (sHR) = 1.47 (95% CI: 1.31‐1.64). However, inclusion of parental history of dementia did not alter the estimates significantly in any of the Fine and Gray models.

**Conclusion:**

There may be substantial heterogeneity among NACC participants with and without parental history of dementia. In our longitudinal analysis, parental history of dementia did not alter the association between depression and conversion to worse cognitive status. This suggests parental history of dementia may lead to differences in participation but may not confound longitudinal associations of interest.

